# Genetic Variability of Morphological, Flowering, and Biomass Quality Traits in Hemp (*Cannabis sativa* L.)

**DOI:** 10.3389/fpls.2020.00102

**Published:** 2020-02-20

**Authors:** Jordi Petit, Elma M. J. Salentijn, Maria-João Paulo, Claire Thouminot, Bert Jan van Dinter, Gianmaria Magagnini, Hans-Jörg Gusovius, Kailei Tang, Stefano Amaducci, Shaoliang Wang, Birgit Uhrlaub, Jörg Müssig, Luisa M. Trindade

**Affiliations:** ^1^Wageningen UR Plant Breeding, Wageningen University and Research (WUR), Wageningen, Netherlands; ^2^Biometris, Wageningen University and Research (WUR), Wageningen, Netherlands; ^3^Fédération Nationale des Producteurs de Chanvre (FNPC), Le Mans, France; ^4^VanDinter Semo BV, Scheemda (VDS), Netherlands; ^5^Centro di ricerca cerealicoltura e colture industriale (CRA), Rovigo, Italy; ^6^Department of Post Harvest Technology, Leibniz-Institute for Agricultural Engineering and Bioeconomy (ATB), Potsdam-Bornim, Germany; ^7^Department of Sustainable Crop Production, Università Cattolica del Sacro Cuore (UCSC), Piacenza, Italy; ^8^The Biological Materials Group, Biomimetics, City University of Applied Sciences Bremen (HSB), Bremen, Germany

**Keywords:** genetic variability, genotype-by-environment (*G*×*E*) interactions, hemp, *Cannabis sativa*, fiber quality, cell wall composition, flowering time, sex determination

## Abstract

Hemp (*Cannabis sativa* L.) is a bast-fiber crop well-known for the great potential to produce sustainable fibers. Nevertheless, hemp fiber quality is a complex trait, and little is known about the phenotypic variability and heritability of fiber quality traits in hemp. The aim of this study is to gain insights into the variability in fiber quality within the hemp germplasm and to estimate the genetic components, environmental components, and genotype-by-environment (*G*×*E*) interactions on fiber quality traits in hemp. To investigate these parameters, a panel of 123 hemp accessions was phenotyped for 28 traits relevant to fiber quality at three locations in Europe, corresponding to climates of northern, central, and southern Europe. In general, hemp cultivated in northern latitudes showed a larger plant vigor while earlier flowering was characteristic of plants cultivated in southern latitudes. Extensive variability between accessions was observed for all traits. Most cell wall components (contents of monosaccharides derived from cellulose and hemicellulose; and lignin content), bast fiber content, and flowering traits revealed large genetic components with low *G*×*E* interactions and high broad-sense heritability values, making these traits suitable to maximize the genetic gains of fiber quality. In contrast, contents of pectin-related monosaccharides, most agronomic traits, and several fiber traits (fineness and decortication efficiency) showed low genetic components with large *G*×*E* interactions affecting the rankings across locations. These results suggest that pectin, agronomic traits, and fiber traits are unsuitable targets in breeding programs of hemp, as their large *G*×*E* interactions might lead to unexpected phenotypes in untested locations. Furthermore, all environmental effects on the 28 traits were statistically significant, suggesting a strong adaptive behavior of fiber quality in hemp to specific environments. The high variability in fiber quality observed in the hemp panel, the broad range in heritability, and adaptability among all traits prescribe positive prospects for the development of new hemp cultivars of excellent fiber quality.

## Introduction

Hemp (*Cannabis sativa* L.) is a well-known bast-fiber crop with evident phenotypic diversity in plant morphology between genotypes. For instance, de Meijer et al. described large diversity in plant height, stem diameter, and stem yield among 206 genotypes. They described accessions up to 4 m high while other plants had a dwarf phenotype with less than 1 m of height (Meijer et al., 1992; Meijer, 1994; Meijer and Keizer, 1996). Extensive diversity has also been described in cannabinoid content, in particular for the major cannabinoids: Δ9-tetrathydrocannabinol (THC) and cannabidiol (CBD) (Meijer et al., 1992; Meijer and Keizer, 1996).

The out-crossing behavior of hemp (Sawler et al., 2015) and its dioecious nature contribute to the variability. Male, female, and monoecious plants are characterized by large sexual dimorphism affecting plant morphology, flowering time, and fiber quality. Male plants have a slender stature, few leaves, flower early, and die after flowering. Moreover, male plants have less lignified cell walls, fine fibers, and large proportion of primary compared to secondary bast fibers. In contrast, female plants are more leafy, flower later, remain alive until seed maturation, accumulate larger content of lignin in the cell walls, and develop larger amount of secondary bast fibers (Amaducci and Gusovius, 2010; Faux et al., 2013). Monoecious plants resemble female plants and are more uniform (Mandolino and Carboni, 2004; Faux et al., 2016). Furthermore, sex determination in cannabis is a quantitative trait. A range of flowers of the opposite sex determined by the genetics can occur in dioecious hemp, and the ratio of female-to-male flowers in monoecious plants is highly variable (Faux et al., 2013; Faux et al., 2014; Faux et al., 2016). A genetic expression analysis between male and female dioecious plants identified nine mRNAs overexpressed in female plants putatively involved in auxin-related gene expression. The study suggested that the repression of female characteristics in male plants implies the downregulation of the genes involved in pathways more strictly related to the differentiation of the female sex (Moliterni et al., 2004). In addition, a range of studies revealed that sex determination of hemp is strongly sensitive to external factors, such as accumulation of Cu^++^, Zn^++^, and Pb^++^ ions or hormonal treatment (Chailakhyan and Khryanin, 1978; Freeman et al., 1980; Soldatova and Khryanin, 2010; Galoch, 2015; Faux et al., 2016). Such studies suggested that in hemp, non-genetic mechanisms, such as epigenetics, might probably affect the control of sex determination (Heikrujam et al., 2014). Consequently, the sexual variation in hemp is expected to be influenced by genetic and environmental components.

Morphological measurements, fiber quality, and flowering traits of hemp respond strongly to environmental factors, particularly to photoperiod and temperature but also to soil composition and crop management (Faux et al., 2013; Amaducci et al., 2015; Sawler et al., 2015). Hemp is a short day plant, and its flowering time is influenced by changes in the photoperiod regime (Amaducci et al., 2012). In locations where the shift from long-day toward short-day photoperiod regimes occurs early, hemp plants flower early, whereas in locations where the shift occurs later, the critical photoperiod for flowering is reached later (reviewed in Salentijn et al., 2019). This behavior affects plant development because plants accumulate biomass during the vegetative growing period, but nutrients are shifted from the production of stem, leaves, and roots toward the production of flowers and seeds around the onset of flowering. In addition, lignification of cell walls intensifies after flowering, along with secondary bast fiber formation (Van Der Werf and Turunen, 2008; Liu et al., 2015). Crop management features such as plant density, irrigation, and harvesting time are also reported to generate differences in phenological traits, such as plant height and stem diameter (Amaducci et al., 2015). Therefore, hemp accessions cultivated under specific environmental conditions are expected to have specific fiber composition and properties.

Hemp is a sustainable fiber crop with great potential for the production of a plethora of bio-based products. Yet, hemp cultivars with improved fiber properties are needed to promote hemp in the emerging bio-based economy. The first step in a breeding program is to characterize the genetic variability for the traits of interest, and that can be done by characterizing them in a wide range of accessions. Understanding the contribution of the genetic (*G*), environment (*E*), and genotype-by-environment (*G*×*E*) interaction components in fiber quality traits is essential to study the stability of fiber quality across different environments and thus improve the success of breeding programs.

To date, little research has been conducted on the variability of hemp traits relevant to fiber quality, such as cell wall composition, stem decortication, bast fiber content after decortication, or fineness of extracted fiber bundles. The objectives of this study are to evaluate the genetic variability, *G*×*E* interactions, and heritability of 28 traits relevant for fiber quality of hemp and identify which traits are worth to be further investigated with mapping studies. The relationships between the 28 traits will also be investigated.

## Materials and Methods

### Plant Material

A test panel of 123 hemp accessions was used in this study to investigate the phenotypic variability of fiber quality in hemp. This panel included mainly fiber accessions, one oil accession, cultivar Finola, one ornamental, and few accessions with other uses (**Table 1**).

**Table 1:** Panel of 123 hemp (*Cannabis sativa* L.) accessions.

MultiHemp code	Accession name/Code	Origin	Accession type	Population type	Provider
MH-AGM-701	Fibrol/Other	Hungary	Fiber	B	AGM
MH-AGM-702	Tiborszallasi/Other	Hungary	Fiber	B	AGM
MH-AGM-703	Tisza/Other	Hungary	Fiber	B	AGM
MH-AGM-704	KC Dora/Other	Hungary	Fiber	B	AGM
MH-AGM-705	Monoica/Other	Hungary	Fiber	B	AGM
MH-CAAS-601	CYM171/Other	China	Fiber	B	CAAS
MH-CAAS-602	CYM28/Other	China	Fiber	B	CAAS
MH-CAAS-603	Yunma 5/Other	China	Fiber	B	CAAS
MH-CAAS-604	CYM49/Other	China	Fiber	B	CAAS
MH-CAAS-605	CYM273/Other	China	Fiber	B	CAAS
MH-CRA-401	CRA_1/Other	Italy	Fiber	B	CRA
MH-CRA-402	CRA_2/Other	Italy	Fiber	B	CRA
MH-CRA-404	Delta llosa/Other	Spain	Fiber	B	CRA
MH-CRA-405	CRA_4/Other	Italy	Fiber	B	CRA
MH-CRA-406	Carma Monoica/Other	Italy	Fiber	B	CRA
MH-CRA-407	Supermono/Other	Italy	Fiber	B	CRA
MH-CRA-408	Fibranova (CRA_5)/Other	Italy	Fiber	B	CRA
MH-CRA-409	Carmagnola/Other	Italy	Fiber	B	CRA
MH-CRA-410	Ermes A/Other	Italy	Fiber	B	CRA
MH-CRA-411	CS (CRA_6)/Other	Italy	Fiber	B	CRA
MH-CRA-412	Carmaleonte/Other	Italy	Fiber	B	CRA
MH-CRA-413	CRA_7/Other	Italy	Fiber	B	CRA
MH-CRA-414	W-1/Other	Italy	Fiber	B	CRA
MH-CRA-415	Zenit/Other	Romania	Fiber	B	CRA
MH-CRA-416	Denise/Other	Romania	Fiber	B	CRA
MH-CRA-417	CRA_8/Other	Italy	Fiber	B	CRA
MH-CRA-418	SVGB-10611/Other	Italy	Fiber	B	CRA
MH-CRA-419	USO 14 Monoica/Other	Ukraine	Fiber	B	CRA
MH-CRA-420	USO 31/Other	Ukraine	Fiber	B	CRA
MH-FNPC-201	Other/A11-121-1	France	Fiber	B	FNPC
MH-FNPC-202	Other/A11-121-2	France	Fiber	B	FNPC
MH-FNPC-203	Other/A11-121-3	France	Fiber	B	FNPC
MH-FNPC-204	Other/A11-121-4	France	Fiber	B	FNPC
MH-FNPC-205	Other/A11-121-5	France	Fiber	B	FNPC
MH-FNPC-206	Other/A11-121-6	France	Fiber	B	FNPC
MH-FNPC-207	Other/A11-121-7	France	Fiber	B	FNPC
MH-FNPC-209	Other/A11-121-9	France	Fiber	B	FNPC
MH-FNPC-210	Other/A11-121-10	France	Fiber	B	FNPC
MH-FNPC-211	Other/A11-121-11	France	Fiber	B	FNPC
MH-FNPC-212	Other/A11-121-12	France	Fiber	B	FNPC
MH-FNPC-213	Other/A11-121-13	France	Fiber	B	FNPC
MH-FNPC-214	Other/A11-121-14	France	Fiber	B	FNPC
MH-FNPC-215	Other/A11-121-15	France	Fiber	B	FNPC
MH-FNPC-216	Other/A11-121-16	France	Fiber	B	FNPC
MH-FNPC-217	Other/A11-121-17	France	Fiber	B	FNPC
MH-FNPC-218	Other/A11-121-18	France	Fiber	B	FNPC
MH-FNPC-219	Other/A11-121-19	France	Fiber	B	FNPC
MH-FNPC-220	Other/A11-121-20	France	Fiber	B	FNPC
MH-FNPC-221	Other/A11-121-21	France	Fiber	B	FNPC
MH-FNPC-222	Other/A11-121-22	France	Fiber	B	FNPC
MH-FNPC-223	Other/A11-121-23	France	Fiber	B	FNPC
MH-FNPC-224	Other/A11-121-24	France	Fiber	B	FNPC
MH-FNPC-225	Other/A10-122-1	France	Fiber	B	FNPC
MH-FNPC-226	Other/A10-122-2	France	Fiber	B	FNPC
MH-FNPC-227	Other/A10-122-4	France	Fiber	B	FNPC
MH-FNPC-228	Other/A103-122-1	France	Fiber	B	FNPC
MH-FNPC-229	Other/A103-122-2	France	Fiber	B	FNPC
MH-FNPC-230	Other/A103-122-3	France	Fiber	B	FNPC
MH-FNPC-231	Other/A103-122-4	France	Fiber	B	FNPC
MH-FNPC-232	Other/A103-122-6	France	Fiber	B	FNPC
MH-FNPC-233	Other/A103-122-8	France	Fiber	B	FNPC
MH-FNPC-234	Other/A103-122-10	France	Fiber	B	FNPC
MH-FNPC-235	Other/A9-122-1	France	Fiber	B	FNPC
MH-FNPC-236	Other/A9-122-2	France	Fiber	B	FNPC
MH-FNPC-237	Other/A9-122-3	France	Fiber	B	FNPC
MH-FNPC-238	Other/A9-122-4	France	Fiber	B	FNPC
MH-FNPC-239	Other/A102-122-1	France	Fiber	B	FNPC
MH-FNPC-240	Other/A102-122-2	France	Fiber	B	FNPC
MH-FNPC-241	Other/A102-122-3	France	Fiber	B	FNPC
MH-FNPC-242	Other/A102-122-4	France	Fiber	B	FNPC
MH-FNPC-243	Other/A102-111-1	France	Fiber	B	FNPC
MH-FNPC-244	Other/A102-111-2	France	Fiber	B	FNPC
MH-FNPC-245	Other/A7-104-1	France	Fiber	B	FNPC
MH-FNPC-246	Other/A7-105-4	France	Fiber	B	FNPC
MH-FNPC-248	Other/B6-093-3	France	Fiber	B	FNPC
MH-FNPC-250	Other/B6-093-17	France	Fiber	B	FNPC
MH-FNPC-251	Férimon/Other	France	Fiber	B	FNPC
MH-FNPC-252	Fédora 17/Other	France	Fiber	B	FNPC
MH-FNPC-253	Félina 32/Other	France	Fiber	B	FNPC
MH-FNPC-254	Epsilon 68/Other	France	Fiber	B	FNPC
MH-FNPC-255	Futura 75/Other	France	Fiber	B	FNPC
MH-FNPC-256	Santhica 27/Other	France	Fiber	B	FNPC
MH-IWNRZ-901	Bialobrzeskie/Other	Poland	Fiber	B	IWNRZ
MH-IWNRZ-902	Beniko/Other	Poland	Fiber	B	IWNRZ
MH-IWNRZ-903	Tygra/Other	Poland	Fiber	B	IWNRZ
MH-LARC-501	Katlakalna/Other	Latvia	Fiber	B	LARC
MH-UOY-801	Finola/Other	Finland	Seed	B	UOY
MH-VDS-301	Chameleon/Other	Netherlands	Fiber	B	VDS
MH-VDS-302	Marcello/Other	Netherlands	Fiber	B	VDS
MH-VDS-303	Markant/Other	Netherlands	Fiber	B	VDS
MH-VDS-304	Ivory/Other	Netherlands	Fiber	B	VDS
MH-WU-101	JSO 16/891229	Russia	Fiber	B	WUR
MH-WU-102	Ajkai-A-TF/891054	Hungary	Fiber	L	WUR
MH-WU-103	Fibrimon 56/880828	France	Fiber	B	WUR
MH-WU-104	Rastislavicke/880816	Slovakia	Fiber	B	WUR
MH-WU-105	Krasnodarskaja 56/891333	Ukraine	Fiber	B	WUR
MH-WU-106	Dneprovskaja 84/921054	Russia	Fiber	L	WUR
MH-WU-107	Other/883290	Russia	Fiber	L	WUR
MH-WU-108	Lovrin 110/883173	Romania	Fiber	B	WUR
MH-WU-109	Bialobrzeskie/891223	Poland	Fiber	B	WUR
MH-WU-110	Other/880973	Spain	Other	Other	WUR
MH-WU-111	Kompolti Sargászáru/883049	Hungary	Fiber	B	WUR
MH-WU-112	Other/883262	Spain	Other	Other	WUR
MH-WU-113	Kompolti hybrid TC/891070	Hungary	Fiber	B	WUR
MH-WU-114	Fibrimon 56/891158	France	Fiber	B	WUR
MH-WU-115	Other/921203	Canada	Other	W	WUR
MH-WU-116	Panorama var. globosa/910914	Hungary	Ornamental	B	WUR
MH-WU-117	Silistrenski/901107	Bulgaria	Fiber	B	WUR
MH-WU-118	Csehslovák-A-TF/891068	Slovakia	Fiber	Other	WUR
MH-WU-119	Other/891288	Poland	Fiber	Other	WUR
MH-WU-120	Other/891090	Turkey	Other	L	WUR
MH-WU-121	Komoroi-A-TF/891046	Hungary	Fiber	L	WUR
MH-WU-122	Other/883289	Russia	Fiber	L	WUR
MH-WU-123	Juznaja Odnovremenno/883293	Russia	Fiber	B	WUR
MH-WU-124	Other/891240	Spain	Other	Other	WUR
MH-WU-125	Orosi-A-TF/891059	Hungary	Fiber	Other	WUR
MH-WU-126	Kompolti/883048	Hungary	Fiber	Other	WUR
MH-WU-127	Dneprovskaja odnodomnaja 6/891326	Ukraine	Fiber	B	WUR
MH-WU-128	Other/891327	Other	Fiber	L	WUR
MH-WU-129	Superfibra/883040	Italy	Fiber	B	WUR
MH-WU-130	Other/891057	Hungary	Other	L	WUR
MH-WU-131	Other/891094	Turkey	Other	L	WUR
MH-WU-132	Other/880817	Germany	Other	Other	WUR

Population types B, L, and W stand for breeding material, landraces, and wild material, respectively. Accession type is based on use. The provider refers to the institution that provided the accessions: AGM, Agromag Kft. from Hungary; CAAS, Bast Fibre Crops-Chinese Academy of Agricultural Science from China; CRA (also known as CREA), Centro di ricerca cerealicoltura e colture industriale from Italy; FNPC, Federation National Producteurs de Chanvre form France; IWNRZ, Institute of Natural Fibres and Medicinal Plants from Poland, LARC; Latgale Agricultural Research Centre from Latvia; UoY, University of York from United Kingdom; VDS, VanDinter Semo from the Netherlands; WU, Wageningen University from the Netherlands. Other stands for no specific accession name, code, origin, accessions type, or population type for those accessions.

### Field Experimental Design

The effects of the environment and the *G*×*E* interactions on the phenotypic variation of fiber quality were assessed on the basis of three locations across Europe at respectively high, mid, and low latitude. The environments mostly differed in photoperiod and temperature regimes and water availability, as shown in **Table 2**. The 123 hemp accessions were grown in: Rovigo, at CRA (Centro di ricerca cerealicoltura e colture industriale) in Italy (45°N 11°E); Chèvrenolles, Neuville-sur-Sarthe, at FNPC (Fédération Nationale des Producteurs de Chanvre) in France (48°N 0.2°E); and Westerlee, at VDS (VanDinter Semo BV) in Netherlands (53°N 6°E). Field trials were performed between April and September 2013. Each field trial had a randomized complete block design with three biological replicates (plots) per accession and location. The experimental units were plots of 1 m^2^ in Italy and Netherlands and of 1.5 m^2^ in France. In all three locations, the same sowing density was used to aim a density of 100 plants/m^2^. Plants in the three middle rows were used for phenotyping. In dioecious accessions, phenotyping was performed only in female plants given the difference in fiber quality due to sex dimorphism in hemp. Field trials were harvested at temperature degree days (∑°C, the cumulated Celsius degree day over a period at a base temperature of 1°C) of 1,740.25°C, 1,421.1°C, and 1,843.3°C in CRA, FNPC, and VDS, respectively, corresponding to full flowering for most accessions in each location (**Table 2**).

**Table 2:** Environmental characteristics of the three field trial locations (CRA, FNPC, and VDS) during the growing season of the MultiHemp project in 2013.

	CRA	FNPC	VDS
Location	Rovigo	Chèvrenolles	Westerlee
Country	Italy	France	Netherlands
Sow dates	April 18–19	May 15	May 6
Harvest dates	July 15–17	July 29–31	September 6–9
Days of growing season	90	77	126
Daylight April 19 (hours)	13:41	13:51	14:14
Daylight May 15 (hours)	14:51	15:10	15:51
Daylight June 15 (hours)	15:37	16:01	16:57
Daylight July 31 (hours)	14:45	15:01	15:43
Daylight August 31 (hours)	13:20	13:28	13:45
Temperature degree days at harvest ∑°C	1,691.2–1,740.25	1,369.25–1,421.1	1,800.05–1,843.3
Average ∑°C/day	19.12	18.22	14.51
∑rainfall (mm)	184.8	195.7	363
Days without rain (%)	43.9	62.5	51.49
Average rainfall/day	2.031	2.509	2.858
Average min RH%/day	45.23	44.9	59.56
Average max RH%/day	93.02	94.39	97.85

Daylight regimes were attained from https://www.timeanddate.com/sun/; ∑°C, accumulated Celsius degrees during the growing season; ∑rainfall, the total amount of rainfall in millimeters during the growing season; RH%, percentage of relative humidity; CRA, Centro di ricerca cerealicoltura e colture industriale from Italy; FNPC, Federation National Producteurs de Chanvre form France; VDS, VanDinter Semo from the Netherlands.

### Phenotypic Data Analysis

In total, 28 parameters were phenotyped, including five agronomic traits, four different flowering traits including sex determination, nine fiber measurements (morphological and processing-related properties), and 10 parameters of cell wall composition (**Table 3**).

**Table 3:** Summary statistics of 27 traits in 123 hemp accessions.

Trait	Abbreviation	Trait group	Cell wall	Mean	Min.	Max.	Range	Standard deviation	Coefficient of variation (CV%)
Stem diameter, after harvest (mm)	D	Agronomic	–	8.776	3.267	24.02	20.76	4.076	46.44
Total DW (dry weight) of five plants as fraction of the FW (fresh weight) of five plants (%)	DW(%)	Agronomic	–	0.358	0.218	0.729	0.511	0.0917	25.64
Dry weight five stems (%)	DW_S(%)	Agronomic	–	0.703	0.332	0.824	0.492	0.0711	10.11
Dry weight five stems (g)	DW_S5(g)	Agronomic	–	146.3	5.4	844.1	838.7	146.6	100.2
Stem height (cm)	H	Agronomic	–	198.5	56.13	324.8	268.7	63.91	32.19
Acid Detergent Lignin (%)	ADL%dm	Cell wall	Lignin	9.071	7.249	13.83	6.58	0.912	10.05
Arabinose (%)	Ara%dm	Cell wall	Pectin	0.797	0.412	1.207	0.794	0.22	27.61
Galactose (%)	Gal%dm	Cell wall	Pectin	1.555	0.924	2.156	1.231	0.311	20.01
Galacturonic acid (%)	GalA%dm	Cell wall	Pectin	4.739	3.372	6.685	3.313	0.781	16.48
Glucose (%)	Glc%dm	Cell wall	Cellulose	48.89	41.73	56.52	14.8	2.584	5.285
Glucuronic acid (%)	GlcA%dm	Cell wall	Xylan	0.367	0.205	0.554	0.349	0.0696	18.98
Klasson Lignin (%)	KL%dm	Cell wall	Lignin	14.7	10.71	19.34	8.627	1.374	9.342
Mannose (%)	Man%dm	Cell wall	Mannan	2.738	1.826	3.773	1.946	0.332	12.12
Rhamnose (%)	Rha%dm	Cell wall	Pectin	0.746	0.619	0.903	0.284	0.0565	7.571
Xylose (%)	Xyl%dm	Cell wall	Xylan	13.63	10.74	17.5	6.761	1.453	10.67
SHIVES% (%)	(χ)	Fiber	–	11.76	0	38.68	39.2	5.806	49.39
Bast content after decortication (%)	BCD%	Fiber	–	29.07	11.61	51.05	39.44	6.431	22.12
The average stem weight (g)	M_0_	Fiber	–	10.3	0.89	38.99	38.99	6.566	63.76
Fiber weight before the separation (g)	MF0	Fiber	–	24.31	0.111	50.7	50.59	8.923	36.71
Fiber weight after the separation (g)	MF1	Fiber	–	21.96	0.374	57.94	57.56	8.587	39.11
Fiber fineness parameter, high compression (mm water)	PH	Fiber	–	8.468	2.979	21.33	18.35	3.074	36.3
Fiber fineness parameter, low compression (mm water)	PL	Fiber	–	14.1	7.819	22.33	14.51	2.799	19.85
Decortication index (%)	ηDec_1	Fiber	–	80.58	38.79	97.37	58.58	11.04	13.7
Decorticability (%)	ηDec_2	Fiber	–	94	78.77	101.1	22.28	3.503	3.727
Beginning flowering time (∑°C)	FL_BEGIN	Flowering	–	1,178	204.8	2329	2124	338	28.7
Full flowering time (∑°C)	FL_FULL	Flowering	–	1,552	416.8	3466	3049	497.9	32.07
Length of vegetative growth period (days)	VEG	Flowering	–	69.75	12	143	131	20.58	29.51

Min. and Max. values correspond to observation of single measurements.

#### Agronomic Measurements

At harvesting time, plants were cut at the base of the stem, and agronomic traits were measured. Stem height (H in centimeters) was measured after harvest. Stem diameter (D in millimeters) was measured at 10 cm above the ground. H and D were phenotyped at three plants per plot, and data were provided as the mean per plot.

Fresh weight was measured in five entire plants per plot including stem and leaves. Thereafter, leaves and stems were separated and dried at 60°C for 48 h. Different dry weights were calculated: dry weight of five stems (DW_5S in grams), dry weight as a fraction of the fresh weight of five stems (DW_S% in percentage), and dry weight as a fraction of the fresh weight of five plants (DW% in percentage).

#### Flowering Parameters

Emergence of the plants was scored as the accumulated Celsius degree days or temperature sum (∑°C) at the day of first emergence. Emergence was scored in one row per plot at day = N, N + 2, N + 4, and N + 7, where N is the day of sowing. Flowering time traits were also measured in ∑°C at 10 plants per plot. Beginning of flowering (FL_Begin in ∑°C) and full flowering (FL_Full in ∑°C) were calculated relative to the emergence as:

(1) $$\text{FL}\_\text{Begin}\;=\;\sum {}^{\circ}{\text{C}}_{\text{Beginning}\;\text{flowering}}\;-\sum {}^{\circ}{\text{C}}_{\text{Emergence}},$$

(2) $$\text{FL}\_\text{Full}\;=\;\sum {}^{\circ}{\text{C}}_{\text{Full}\;\text{flowering}}\;-\sum {}^{\circ}{\text{C}}_{\text{Emergence}},$$

where ∑°C_Beginning_ _flowering_, ∑°C_Full_ _flowering_, and ∑°C_Emergence_ are the accumulated Celsius degree days, respectively, at the beginning of flowering, full flowering, and at the day of first emergence. The length of vegetative growth period (VEG in days) is the growing period of the plants in days, as measured from the day of first emergence until FL_Begin. Sex determination was phenotyped assessing “1” for predominantly dioecious plants, “2” for the mix of dioecious and monoecious plants, and “3” for predominantly monoecious.

#### Fiber Traits (Morphological and Processing-Related Properties)

The measurements of the processing-related properties were performed on stem segments of at least 100 cm, discarding 20 scm from the base of the plant and removing 30 cm from the top. Stem portions were naturally dried, in open air under a roof, until the water content was less than 18% of the mass. Thereafter, stems were warm water retted for 3 days at an average temperature of 23°C according to (Van Den Oever et al., 2003). After water retting, stems were naturally dried again and stored at 20°C ± 3°C and relative humidity of 60% ± 5%. All stem weight measurements were calculated as an average in grams of 10 stems. The first measurement of stem weight, M_0_, was performed straight before the decortication. Each specimen was decorticated individually with a lab-scale roller-breaker decortication system according to Wang et al. (2018). Stem portions passed through all decortication steps six times. The weight of each decorticated specimen was measured and recorded after each passage through the decorticator, M_i_, in grams, where “i” is the passage number from 1 to 6. After the sixth passage, the remaining shives [also known as woody hemp core (WHC)] were removed manually from the bast, and the shives-free bast was weighed (M_7_). The fiber bundles of the shive-free bast were separated using a Worthmann coarse separator unit (Worthmann Maschinenbau GmbH, Barßel-Harkebrügge, Germany). The weight of the separated fiber bundles were measured and recorded before (MF0 in grams) and after (MF1 in grams) the separation.

The stem weight (M_0_) as well as the remaining weights after respective decortication steps (M_2_, M_6_, and M_7_) were used to calculate the bast content and the decortication efficiency parameters, according to Wang et al. (2018). Bast content after decortication (BCD in percentage) was calculated as the fraction between the mass of shives-free bast (M_7_) and the mass of the initial non-decorticated stems (M_0_):

(3) $$BCD\%=\;\frac{M_{7}}{M_{0}}\;\times \;100.$$

The initial decortication efficiency (η_Dec_1_ in percentage) describes the efficiency of the initial stage of the decortication process. It was calculated by using the following formula:

(4) $$n_{Dec\_1}=\frac{M_{0}-M_{2}}{M_{0}-M_{7}}\;\times \;100.$$

The ultimate decortication efficiency (η_Dec_2_ in percentage) estimates the efficiency of the overall decortication process known as decorticability. The decorticability indicates the difference between the weight of the bast fiber after the final removal of the remaining shives after the decortication (M_7_) and the weight of the bast after the last round of the decortication process (M_6_):

(5) $$n_{Dec\_2}=\frac{M_{0}-M_{6}}{M_{0}-M_{7}}\;\times \;100.$$

Shives content after decortication (χ in percentage) describes the ratio of the shives that remained stuck to the bast after the decortication:

(6) $$\left(\chi \right)=\frac{M_{6}-M_{7}}{M_{6}}\;\times \;100.$$

Fineness of extracted fiber bundles was indirectly characterized by measuring the permeability of air flow injected in the bast fiber bundles with a defined mass (Müssig and Amaducci, 2018). The permeability of air is an indicator of the fiber bundle surface. Fineness was measured using a Shirley IIC Fineness and Maturity Tester (Shirley FMT) according to Müssig (2001) and Müssig et al. (2010). Twelve technical replicates of 4 ± 0.005 g of separated bast fiber bundles were weighed for the analysis after 24 h of acclimatization at 20°C and 65% relative humidity of air for sample standardization. Two different air compressions were injected in each sample: low compression of air at a flow rate of 4 L of air per minute and high compression at a flow rate of 1 L of air per minute (Montalvo and Faught, 1999). Two different Shirley values were obtained: P_L_ and P_H_. P_L_ (in millimeter water) is the pressure of the air injected at a low compression and P_H_ (in millimeter water) is the pressure of the air injected at a high compression of air. Both measurements were calculated as the mean of the twelve specimens per sample.

#### Biochemical Analysis of Hemp Cell Walls

Hemp cell walls are mostly composed of polysaccharides and lignin, and this was therefore the main target of the biochemical analysis. Polysaccharide composition was measured based on the content of the monosaccharides that are specific for each polysaccharide. In total, 10 cell wall parameters were measured: the monosaccharide glucose (Glc%dm) that is mostly composing cellulose; mannose (Man%dm) composing mannan; xylose (Xyl%dm) and glucuronic acid (GlcA%dm) composing xylan; arabinose (Ara%dm), galactose (Gal%dm), galacturonic acid (GalA%dm), and rhamnose (Rha%dm) composing pectin, and furthermore two measurements for lignin, Klasson lignin (KL%dm) and acid detergent lignin (ADL%dm). All parameters were calculated as percentage of the dry matter. All cell wall traits were measured with multivariate prediction models based on near-infrared spectroscopy (NIRS), after a calibration curve for the 10 different cell wall traits in hemp was developed. In detail, five stems of each plot were harvested, after which the un-retted stem were dried, pooled and grinded according to Petit et al. (2019) and scanned using a Foss DS2500 near-infrared spectrometer (Foss, Hillerød, Denmark) to obtain the NIR spectra of stem samples [details in (Van Der Weijde et al., 2016)]. A subset of 114 samples was selected based on the variation of the NIR spectra and biochemically analyzed (Petit et al., 2019) to develop the prediction models. Details of the quality of the models can be found in **Supplementary Table 1** and **2**.

### Statistical Analyses

In order to study the variability of fiber quality in hemp, an ANOVA model was used to determine the significant differences of each variance component in the 28 traits: genotype (*G*), environment (*E*), blocks within environment (*B*), *G*×*E* interactions, and residual variance (*ϵ*). The analysis was performed following the model:

(7) y=μ+E+B+G+G×E+ϵ,

where *y* is the trait, *µ* is the grand mean, *E* is the effect due to the environment, *B* is the effect of block within environment, *G* is the genotypic effect, *G*×*E* is the genotype-by-location interactions, and *ϵ* is the residual error. In addition, a random effects model was used to determine the estimates of variance components of the phenotypic variation following the model:

(8) $$\sigma_{y}^{2}=\sigma_{E}^{2}+\sigma_{B(E)}^{2}+\sigma_{G}^{2}+\sigma_{G\times E}^{2}+\sigma_{\epsilon }^{2},$$

where *σ_y_^2^*, *σ_E_^2^*, *σ_B_^2^*, *σ_G_^2^*, *σ_G_*_×_*_E_^2^*, *σ_ϵ_^2^* are the variances for *y*, *E*, *B*, *G*, *G*×*E*, and *ϵ*, respectively. The variance components were reported as the percentage of each component to the total phenotypic variation. Both ANOVA and random effects models were performed using a restricted maximum likelihood (REML) algorithm. For each trait, the stability of the accessions across locations was determined with the size of the variation due to *G*×*E* interactions relative to the main genotypic component, as in (Gitonga et al., 2014):

(9) $$Ratio\;GxE/G\;=\;\frac{\sigma_{G\times E}^{2}}{\sigma_{G}^{2}}.$$

The broad-sense heritability values (H^2^) were calculated across the three environments, as the fraction of the genetic component ($\sigma_{G}^{2}$) to the total genotypic effect ($\sigma_{G}^{2}$, $\sigma_{GxE}^{2}$, and $\sigma_{\epsilon }^{2}$) including the *G*×*E* interactions and the residual variance corrected by the number of blocks and environments, as in Renaud et al. (2014):

(10) $$H^{2}\;=\;\frac{\sigma_{G}^{2}}{\sigma_{G}^{2}+\frac{\sigma_{G\times E}^{2}}{n\cdot E}+\frac{\sigma_{\epsilon }^{2}}{n\cdot B\;\times n\cdot E}}.$$

Where *n*·*E* is the number of environments, and *n*·*B* is the number of blocks. REML and broad-sense heritability (H^2^) analyses were performed using Genstat 19th edition software (VSN International, Hemel Hempstead, UK).

Summary statistics of the 28 traits and the accessions was performed in Genstat 19th edition software. Correlation analysis between the 28 traits was performed in R (http://www.r-project.org/) version 3.4.3 statistical software using corrplot function. The adjusted mean of the phenotypic values across the three locations was used for each trait to study the main correlations independently of the effect of the environment.

## Results

### Fiber Quality Variability of the Hemp Accession Panel

Significant differences between the averaged performance across the three environments for all accessions were found for all traits (*p < 0.001*). Most traits showed extensive variation among the accessions of the hemp panel, as revealed by the wide range and the large coefficients of variation for each trait presented in **Table 3**. Traits with wide variation between accessions included Glc%dm, Man%dm, Xyl%dm, ADL%dm, KL%dm, BCD%, P_H_, P_L_, and four flowering traits (**Table 3**). **Tables 4**–**6** show the averaged phenotypic values and the coefficients of variation across all environments of these 12 traits for the accessions that displayed the most contrasting phenotypic values.

**Table 4:** Summary statistics of hemp accessions with extreme phenotypes for five cell wall traits.

Accession	ADL%dm		Glc%dm		KL%dm		Man%dm		Xyl%dm	
	Mean ± SD	CV%	Mean ± SD	CV%	Mean ± SD	CV%	Mean ± SD	CV%	Mean ± SD	CV%
**MH_IWNRZ_902**	7.72 ± 0.20	(2.63)	54.50 ± 2.32	(4.26)	11.93 ± 0.48	(4.02)	3.29 ± 0.42	(12.74)	11.36 ± 0.65	(5.52)
MH_LARC_501	10.89 ± 0.83	(7.61)	44.57 ± 0.64	(1.43)	16.93 ± 1.48	(8.75)	2.34 ± 0.35	(14.88)	15.83 ± 1.45	(9.17)
MH_UOY_801	12.81 ± 0.89	(6.92)	43.00 ± 1.33	(3.08)	18.18 ± 0.34	(1.88)	2.19 ± 0.26	(11.94)	15.87 ± 1.09	(6.84)
**MH_WU_111**	8.03 ± 0.73	(9.05)	53.58 ± 2.67	(5.01)	12.08 ± 1.44	(11.92)	2.65 ± 0.20	(7.63)	11.68 ± 0.99	(8.48)
MH_WU_122	11.78 ± 0.71	(6.02)	43.95 ± 0.80	(1.83)	17.63 ± 0.71	(4.04)	2.28 ± 0.35	(15.47)	15.64 ± 1.62	(10.34)

Phenotypic values indicate the averages across the three environments. Promising accessions across all three locations are highlighted in bold. See **Table 3** for abbreviations.

**Table 5:** Summary statistics of hemp accessions with extreme phenotypes for three fiber traits.

Accession	BCD%		PH		PL	
	Mean ± SD	CV%	Mean ± SD	CV%	Mean ± SD	CV%
MH_CAAS_601	16.68 ± 4.42	(26.52)	14.62 ± 6.21	(42.46)	19.69 ± 3.90	(19.82)
**MH_IWNRZ_902**	41.50 ± 10.89	(26.24)	3.40 ± 0.60	(17.48)	8.69 ± 1.23	(14.14)
MH_LARC_501	21.74 ± 3.76	(17.32)	13.81 ± 2.70	(19.45)	17.41 ± 0.77	(4.39)
MH_UOY_801	17.89 ± *	(*)	*	(*)	*	(*)
**MH_WU_111**	40.70 ± 2.36	(5.80)	6.70 ± 1.68	(25.90)	12.76 ± 1.38	(10.81)

Phenotypic values indicate the averages across the three environments. Promising accessions across all three locations are highlighted in bold. (*) stands for missing value. See **Table 3** for abbreviations.

**Table 6:** Summary statistics of hemp accessions with extreme phenotypes for flowering traits.

Accession	FL_Begin		FL_Full		VEG		Sex_det	
	Mean ± SD	CV%	Mean ± SD	CV%	Mean ± SD	CV%	Mean	CV%
MH_CAAS_601	2,057.40 ± 470.42	(22.87)	3,428.50 ± 64.95	(1.89)	119.00 ± 35.68	(29.98)	1 ± 0.00	(0.00)
MH_CAAS_602	2,071.60 ± 445.83	(21.52)	3,442.53 ± 40.65	(1.18)	119.57 ± 34.70	(29.02)	1 ± 0.00	(0.00)
MH_CAAS_603	2,087.42 ± 389.66	(18.67)	3,440.87 ± 43.53	(1.26)	117.78 ± 30.76	(26.12)	1 ± 0.00	(0.00)
MH_CAAS_604	1,928.48 ± 418.76	(21.71)	2,663.01 ± 705.98	(26.51)	106.92 ± 33.36	(31.20)	1 ± 0.00	(0.00)
MH_CAAS_605	2,090.60 ± 411.36	(19.68)	3,440.87 ± 43.53	(1.26)	115.92 ± 31.00	(26.75)	1 ± 0.00	(0.00)
MH_CRA_415	784.87 ± 131.15	(16.71)	1,059.09 ± 124.30	(11.74)	50.51 ± 12.30	(24.34)	2.44 ± 0.51	(20.83)
MH_IWNRZ_901	929.90 ± 190.08	(20.44)	1,246.96 ± 129.77	(10.41)	57.95 ± 17.05	(29.41)	3 ± 0.00	(0.00)
**MH_IWNRZ_902**	997.02 ± 214.51	(21.52)	1,293.58 ± 97.08	(7.50)	61.23 ± 18.01	(29.42)	3 ± 0.00	(0.00)
MH_IWNRZ_903	951.50 ± 146.35	(15.38)	1,287.25 ± 31.05	(2.41)	58.50 ± 12.55	(21.46)	3 ± 0.00	(0.00)
MH_LARC_501	620.85 ± 236.13	(38.03)	840.68 ± 248.70	(29.58)	41.15 ± 20.45	(49.71)	1.22 ± 0.38	(31.49)
MH_UOY_801	469.03 ± 239.07	(50.97)	639.83 ± 204.32	(31.93)	32.63 ± 20.16	(61.78)	1 ± 0.00	(0.00)
MH_WU119	685.38 ± 347.01	(50.63)	965.48 ± 342.91	(35.52)	47.68 ± 29.53	(61.93)	1.83 ± 0.24	(12.86)
MH_WU_122	440.12 ± 165.34	(37.58)	615.45 ± 148.60	(24.14)	30.34 ± 14.38	(47.40)	1 ± 0.00	(0.00)

Phenotypic values indicate the averages across the three environments. Promising accessions across all three locations are highlighted in bold. See **Table 3** for abbreviations. Sex_det stands for Sex determination.

Accessions IWNRZ-902 (Beniko) and WU-111 (Kompolti Sargászáru) showed the largest contents of Glc%dm, Man%dm, and BCD% while LARC-501 (Katlakalna) and UOY-801 (Finola) showed the opposite phenotypic characteristics. In contrast, the opposite patterns were found for Xyl%dm, ADL%dm, and KL%dm where IWNRZ-902 and WU-111 showed the lowest phenotypic values and LARC-501 and UOY-801 showed the largest values (**Tables 4** and **5**). CAAS-601 showed the finest fiber bundles while IWNRZ-902 showed the coarsest ones, as presented in **Table 5**. Chinese accessions (CAAS) were the latest to flower and showed the longest vegetative growth period (VEG). In addition, some Chinese accessions (CAAS-601, CAAS-602, CAAS-603, and CAAS-605) did not reach full flowering, before the end of the field trials, in Netherlands and France but they did in Italy (data not shown). In contrast, LARC-501, UOY-801, and WU-122 were the earliest accession to flower and to reach full flowering, and they showed the shortest VEG (**Table 6**). Finally, contrasting accessions for sex determination can be found in **Table 6**. Sex determination highlighted large range of variation between predominantly dioecious and predominantly monoecious. For instance, Chinese accessions and UOY-801 showed only dioecious plants (score = 1), and IWNRZ-901, IWNRZ-902, and IWNRZ-903 showed only monoecious plants (score = 3) while other accessions showed dioecious plants mixed with monoecious plants in different proportions. LARC-501 showed larger number of dioecious than monoecious plants (score = 1.22), WU-119 showed approximately equal amount of dioecious and monoecious plants, while CRA-415 showed more monoecious than dioecious plants (score = 2.44).

### Elucidating the Key Components of Fiber Quality Variability in the Hemp Panel

The ANOVA model highlighted significant differences (*p < 0.001*) for all variance components in all traits. In addition, as shown in **Table 7** and **Supplementary Figure 1**, the random effects model revealed traits with phenotypic variations strongly influenced by the genetic component and traits mostly influenced by the environment component.

**Table 7:** Variance components and broad-sense heritability (H^2^) of 28 traits calculated with a random effects model.

Trait	Location (*L*%)	Block within Location (*B*%)	Genotype (*G*%)	Genotype × Location (*LG*%)	Error (*ϵ*%)	Ratio *G*×*E/G*	H^2^
D	84.13	1.72	3.16	1.00	9.98	0.32	0.69
DW(%)	69.41	4.43	0.89	2.70	22.57	3.03	0.21
DW_S(%)	14.08	1.45	31.15	13.01	40.30	0.42	0.78
DW_S5(g)	60.37	4.63	1.96	1.30	31.74	0.66	0.33
H	81.81	1.33	6.84	2.31	7.71	0.34	0.81
ADL%dm	22.52	0.98	44.14	8.72	23.64	0.20	0.89
Ara%dm	86.87	0.59	1.35	1.92	9.26	1.42	0.45
Gal%dm	85.38	1.16	3.30	2.16	8.00	0.65	0.67
GalA%dm	80.77	0.38	4.54	2.88	11.44	0.63	0.67
Glc%dm	11.14	1.52	67.57	4.30	15.48	0.06	0.96
GlcA%dm	46.64	1.96	29.77	2.97	18.66	0.10	0.91
KL%dm	34.04	0.45	47.73	3.31	14.47	0.07	0.95
Man%dm	21.24	0.53	41.62	8.24	28.36	0.20	0.88
Rha%dm	81.54	0.66	5.31	2.71	9.78	0.51	0.73
Xyl%dm	23.77	0.29	54.79	4.86	16.29	0.09	0.94
(χ)	8.91	13.73	11.97	19.22	46.16	1.61	0.51
BCD%	16.66	0.89	66.79	5.36	10.29	0.08	0.96
M_0_	64.98	0.00	1.88	7.60	25.54	4.04	0.26
MF0	27.39	9.67	2.68	22.59	37.66	8.43	0.19
MF1	23.37	6.51	0.61	18.73	50.78	30.70	0.05
PH	62.47	0.80	15.03	5.61	16.09	0.37	0.80
PL	50.25	1.18	14.89	12.59	21.09	0.85	0.69
ηDec_1	67.25	1.28	2.30	12.45	16.71	5.41	0.28
ηDec_2	26.14	11.10	5.29	16.61	40.87	3.14	0.34
FL_Begin	9.06	0.40	74.43	8.97	7.15	0.12	0.95
FL_Full	4.62	0.05	78.86	14.66	1.81	0.19	0.94
VEG	42.44	0.30	46.13	6.16	4.98	0.13	0.95
Sex_det	2.53	0.91	68.90	10.52	17.13	0.15	0.93

The variances explained by each component are shown as the proportion of total variance (%). All components of the phenotypic variation showed significant differences (*p < 0.001*) in each trait, calculated with an ANOVA model. See **Table 3** for abbreviations. Sex_det stands for Sex determination.

Traits with extensive influence of the genetic component (>40%) comprised flowering traits, cell wall traits including contents of monosaccharides derived from cellulose and hemicelluloses, lignin content, and the fiber trait BCD% (**Table 7** and **Supplementary Figure 1**). The variation in flowering traits: FL_Begin, FL_Full, VEG, and Sex_det showed genetic components of respectively 74%, 79%, 46%, and 69%; the content of the monosaccharide from cellulose, Glc%dm, was 68%, and the contents of monosaccharides from hemicelluloses were calculated respectively 55% (for Xyl%dm) and 42% (for Man%dm). The two measurements of lignin displayed genetic components of 44% (for ADL%dm) and 48% (for KL%dm), respectively. BCD% showed a genetic component of 67%. All these 10 traits showed larger genetic component than *G*×*E* interaction. Ratios *G*×*E/G* close to zero were detected for all these traits indicating large stability of the accession ordering across environments. Consequently, all these traits displayed high H^2^, ranging from 0.88 to 0.96, as detailed in **Table 7**.

Traits with large influence of the environment component (>30%) comprised several agronomic traits, cell wall traits such as the content of monosaccharides composing pectin and GlcA%dm, and most fiber traits (**Table 7** and **Supplementary Figure 1**). Agronomic traits such as D, H, DW(%), and DW_S5(g) showed environment components larger than 60%. The composition of pectin, reflected by the contents of Ara%dm, Gal%dm, GalA%dm, and Rha%dm, was extensively influenced by the environment component (>80%). Glucuronic acid, a component of xylan (hemicellulose), expressed as GlcA%dm, showed a strong influence of the environment (47%) but also highlighted a substantial genetic component (30%). In addition, fiber traits such as η_Dec_1_, P_H_, P_L_, and M_0_ showed environment components of respectively 67%, 62%, 50%, and 65%. The ratios *G*×*E/G* showed different performances in these agronomic measurements, fiber traits, and pectin-related monosaccharides. DW(%), Ara%dm, M_0_, and η_Dec_1_ showed large *G*×*E/G* ratios ranging from 1.42 to 5.41. These traits showed large differences between accessions in environmental sensitivity, indicating alteration of the accession ordering across environments. These differences in sensitivity were reflected by low H^2^, ranging from 0.21 to 0.45. In contrast, D, DW_S5(g), H, Gal%dm, GalA%dm, Rha%dm, P_H_, and P_L_ showed interaction ratios ranging from 0.32 to 0.85. These results may indicate that, despite the significant genetic component of the phenotypic variation, the small effects of the genetic component in some traits [particularly D, DW_S5(g), H, Gal%dm, GalA%dm, and Rha%dm] hampered the assessment of the *G*×*E%* interactions, and thus the ratios *G*×*E/G* are small. As a consequence, considering the definition of H^2^ (Renaud et al., 2014), the ratios *G*×*E/G* below 1 can explain unexpected H^2^ (Gitonga et al., 2014), ranging from 0.33 to 0.8, from traits with mostly environment component.

### Large Adaptive Behavior of Hemp Fiber Quality Under Specific Environments

**Figure 1:**
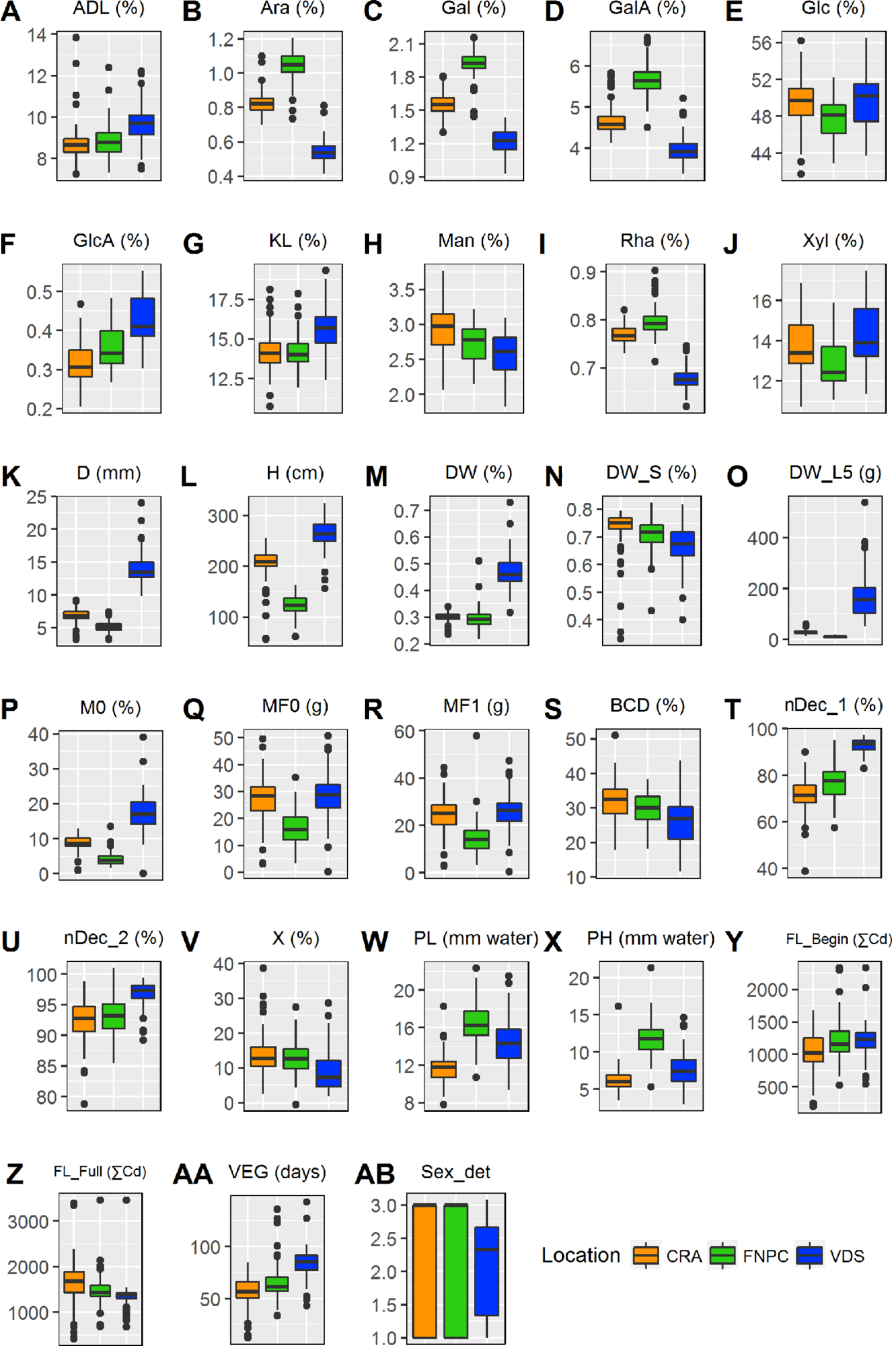
Box plots summarizing the variation of a hemp panel for 28 diverse traits in three locations with contrasting environments. For every box plot, the horizontal line represents the median of the trait, the box represent the interquartile range, the bars outside the box represent the extremes and the crosses indicate the outliers. In every panel, the x-axis indicates the location, as specified in the legend. Range of statistical differences across locations are available in **Supplementary Table 3**. See **Table 3** for abbreviations. Sex_det stands for Sex determination.

The significant effect of the environment component of all traits suggested strong adaptability of hemp fiber quality to different environmental conditions. **Figure 1** shows environmental specific responses or adaptations of these traits in different locations. Plants grown in Netherlands were quite different from plants grown in the other two locations. They produced larger biomass [DW(%), DW_S5(g), lignin content, and GlcA%dm], thicker stems, taller plants, and plants flowered later and over a shorter period than in the other locations. In addition, the decortication parameters [ηDec_1, ηDec_2, and (χ)] showed larger efficiencies in stems from plants grown in this location. In contrast, monosaccharides composing pectin showed the largest contents in plants grown in France while the lowest contents were found in plants grown in Netherlands. Fineness properties followed the same pattern as pectin-related monosaccharides. Finally, plants grown in Italy flowered earlier and over a longer period of time.

### Elucidating the Relationships Between Traits Relevant to Hemp Fiber Quality

**Figure 2:**
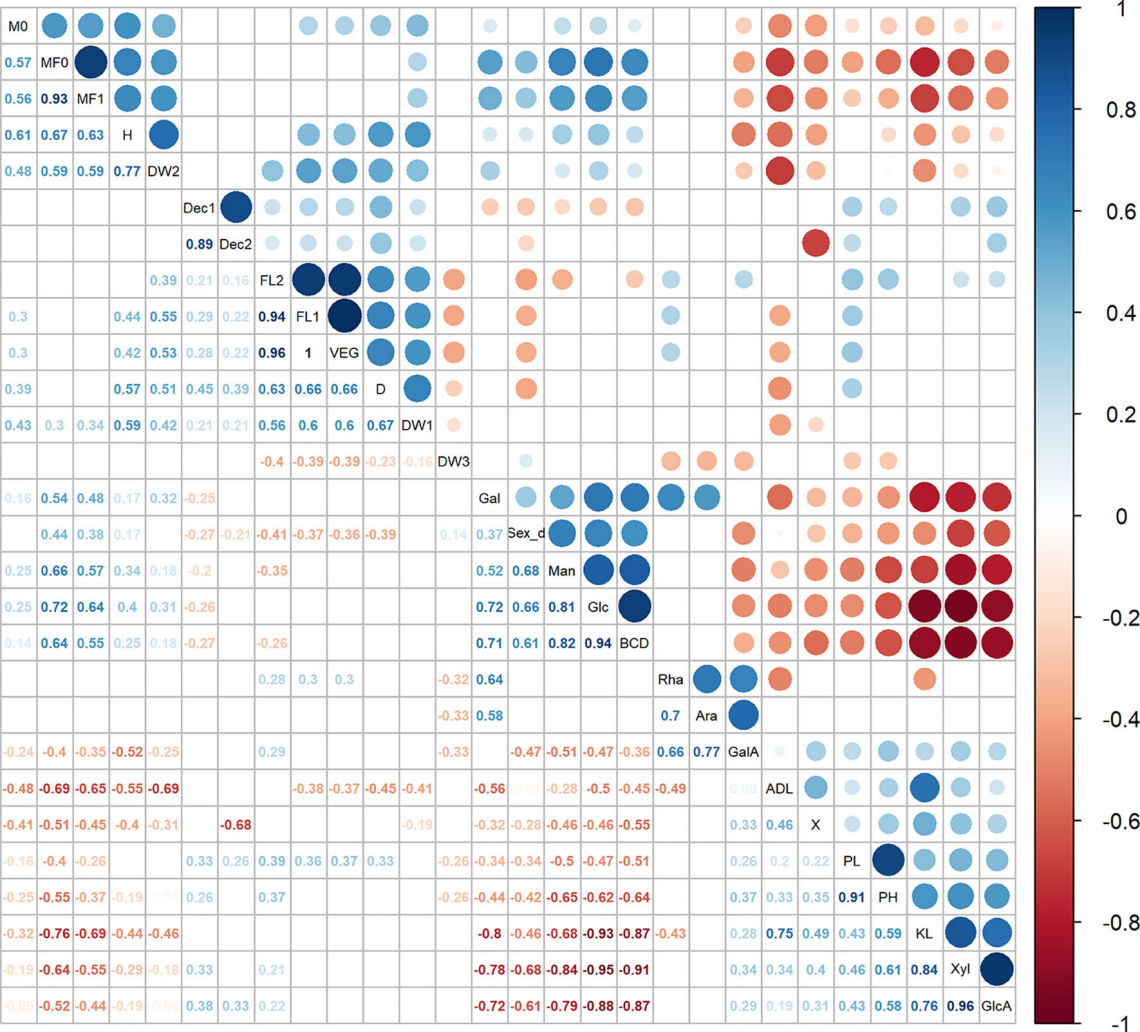
Correlation analysis between 28 agronomic measurements, flowering stages, fiber traits, and cell wall components. Significant correlations were set at a confidence level of 0.95, and blank cells represent no significant correlations. Rha, Rha%dm; Ara, Ara%dm; Gal, Gal%dm; GalA, GalA%dm; Glc, Glc%dm; Man, Man%dm; Xyl, Xyl%dm; GlcA, GlcA%dm; KL, KL%dm; ADL, ADL%dm; FL1, FL_Begin; FL2, FL_Full; Sex_d, Sex determination; DW1, DW_S5(g); DW2, DW_S(%); DW3, DW(%); BCD, BCD%; X, (χ); Dec1, ηDec_1; Dec2, ηDec_2. See **Table 3** for abbreviations.

The fiber trait BCD% showed strong correlations with cell wall components, as detailed in **Figure 2** and **Supplementary Figure 2**. BCD% was positively correlated with Glc%dm and Man%dm (respectively r^2^ = 0.94 and r^2^ = 0.82) and negatively correlated with Xyl%dm and GlcA%dm (respectively r^2^ = -0.91 and r^2^ = -0.87). In addition, the decortication trait (χ) showed a small but significant positive correlation with GalA%dm (r^2^ = 0.33). The contents of lignin (KL%dm) and Glc%dm highlighted the largest negative correlation (r^2^ = -0.93). Lignin measurement ADL%dm was negatively correlated to the fraction of the total dry weight derived from stems [DW_S(%)] and to flowering time traits. Flowering time traits were positively correlated to D and DW_S5(g). Finally, sex determination was positively correlated to the BCD%, Glc%dm, and Man%dm while it was negatively correlated to the contents of lignin (KL%dm), Xyl%dm, GlcA%dm D and flowering time traits. The positive correlations with sex determination were associated to monoecious accessions or accessions with a larger fraction of monoecious plants while the negative correlations were associated to dioecious accessions or accessions with a larger fraction of dioecious plants.

## Discussion

### Fiber Quality Traits Are Extensively Diverse and Heritable but Also Adaptable to Specific Environments

In the present study, a panel of 123 hemp accessions was used to study the variability in hemp fiber quality and to enlighten key components of this variability. The analysis of the hemp panel revealed extensive variation in 28 fiber quality-related traits among the hemp accessions. In addition, some accessions displayed characteristics that are highly appreciated by the hemp industry. Such traits included large contents of bast fiber and cellulose, low contents of lignin and pectin, fine fiber bundles (high P_L_ and P_H_ values), and late flowering time (Ranalli, 2004; Salentijn et al., 2015). Accessions IWRNZ-902 and WU-111 exhibited several of these phenotypes and thus have a considerably higher quality fiber compared to many other accessions. These accessions indicate valuable germplasm to include in breeding programs, aiming to improve hemp fiber quality.

We observed that important fiber quality traits have a large fraction of heritable phenotypic variation, as indicated by the large *G*% and H^2^. As in the present study, extensive genetic studies have shown large heritability values for cell wall components (cellulose, hemicellulose, and lignin) in other fiber crops, such as poplar and eucalyptus (Raymond and Schimleck, 2002; Klasnja et al., 2003; Schimleck et al., 2004; Poke et al., 2006; Davis, 2008), miscanthus (Slavov et al., 2014; Van Der Weijde et al., 2017), switchgrass (Mclaughlin et al., 2006; Boe and Lee, 2007), and maize (Torres et al., 2015). Furthermore, similar heritability values for flowering time were reported in several plant species such as almond [reviewed in Sánchez-Pérez et al. (2014)], apricot (Campoy et al., 2011), arabidopsis (Sasaki et al., 2015), cotton (Kushanov et al., 2017), flax (Soto-Cerda et al., 2014; You et al., 2017), and rice (Takahashi et al., 2001; Huang et al., 2010). It seems plausible that a large fraction of the phenotypic variation of biomass and flowering traits might be controlled by highly “robust genetic systems,” although they are highly complex and polygenic traits, since respectively ~4,000 (Wang et al., 2012) and ~300 genes are estimated to be involved in cell wall synthesis and flowering in arabidopsis (Wang et al., 2012; Bouché et al., 2016). The robust genetic systems might work to control the performance of these traits so that they are less sensitive to environmental differences. This guarantees important functions such as fiber production and reproduction regardless of the environment.

The crucial functions of these traits might support such high heritability values controlled by robust genetic mechanisms. From an evolution point of view, the cell wall performs a structural function in shaping the cells and consequently to plant bodies (Sarkar et al., 2009). Particularly, cellulose and lignin can withstand mechanical pressure exerted by the gravitational pull and the load of the plant body, providing mechanical strength to the plant (Volkmann and Baluška, 2006). In addition, lignin provides protection functions against UV radiation and against pathogens, such as microbes, fungi, and animals, that allowed plants to conquer terrestrial habitats (Iiyama et al., 1994; Popper et al., 2011). Hemicellulosic polysaccharides also provide structural rigidity to the cell walls (Pauly et al., 2013). Hemicellulose and lignin create a matrix around microfibrils of cellulose affecting the recalcitrance of the cell walls (Torres et al., 2015). Furthermore, flowering is an essential biological process for many plants as the survival of the species depends on it (Mouradov et al., 2002). Consequently, these biomass and flowering traits seem to perform essential roles that cannot be widely modified, as the consequences might be lethal for the plant.

Furthermore, we observed that hemp fiber quality-related traits are not strongly stable across environments, as indicated by the significance of *G*×*E* interaction components. The results showed that some accessions were more sensitive in some environments than in others. It seems likely that the phenotypic variation associated to the *G*×*E* interactions might be controlled by “plastic genetic systems” where certain genes are expressed when combined with specific environmental conditions. This is the first study describing significant *G*×*E* interactions in fiber and flowering traits in hemp. Studying these interactions is important owing to their implications for the setup of selection experiments, as the ranking of accessions is dependent on the environment (Van Der Weijde et al., 2017). Selection for traits with large *G*×*E* interactions in breeding programs might lead to biased selection decisions, owing to the unknown effects in untested environments. Nonetheless, the *G*×*E* interactions were small in most important cell wall components (contents of glucose, mannose, xylose, ADL, and KL), fiber content, and flowering traits in hemp. Similar small *G*×*E* interactions have been shown for biomass traits, especially contents of cellulose, hemicellulose, and lignin, in several fiber crops, such as alfalfa (Sheaffer et al., 1998), maize (Dolstra et al., 1992; Cox et al., 1994; Argillier et al., 1997; Barrière et al., 2008; Torres et al., 2015), miscanthus (Van Der Weijde et al., 2017), and switchgrass (Hopkins et al., 1995). Therefore, the extent of the *G*×*E* interaction effects on these fiber quality-related traits might not strongly affect the ordering of hemp accessions across environments and might not interfere in selection decisions.

Interesting examples of large *G*×*E* interactions in hemp are the contents of some pectin-related monosaccharides. The evolution and the functions of pectin in plants might explain these results. In the stem of plants, pectins are mostly present in the middle lamella between cells and are involved in the intracellular adhesion, providing integrity and rigidity to plant tissues and to the stem. They also play important roles in the defence mechanisms against pathogens. In addition, they are involved in the regulation of the ion transport and in the water holding capacity (Voragen et al., 2009). Yet, pectic polysaccharides are highly dynamic structures, and their content dramatically changes across tissues and plant species (Willats et al., 2001). Pectin has almost disappeared in the stems from several modern plants, such as grasses (Carpita and Gibeaut, 1993; Carpita, 1996; Sarkar et al., 2009; Voragen et al., 2009), suggesting that their important functions might be evolutionary replaced by other cell wall components. Lignin is the newest cell wall component to appear in plants and has some parallel functions with pectin, such as structural support and defence functions (Sarkar et al., 2009). As a consequence, dramatic changes of pectin content might not be lethal to plants, owing to a putative partial compensation from other cell wall components, which might allow larger plasticity in sensitivity of certain accessions in different environments.

Fiber quality-related traits were strongly influenced by the differences of the environments across the trial locations. Previous studies have reported large sensitivity of hemp to the environment, particularly to the photoperiod and temperature regimes, affecting the vegetative growth and flowering of the plants (Faux et al., 2013; Amaducci et al., 2015; Sawler et al., 2015). This large sensitivity can be understood as a strong general response of hemp accessions to adapt to the environment, independently of the heritable genetic control of the traits described in previous paragraphs. This behavior might be the result of the optimization of the plant fitness under specific environmental conditions. Consequently, the environment of the growing locations should be taken into account when selecting the cultivation purpose of hemp, and subsequent breeding should be done for use in a specific environment.

An example of the adaptive behavior of hemp is the difference in biomass production and flowering time across environments. Plants of the same accession grown in the Netherlands produced larger biomass and flowered later than plants grown in the other two locations. These variations can be explained by differences in photoperiod regimes across locations. Hemp is a short-day plant, and the length of the vegetative growth period depends on the shift from long- to short-day photoperiod regimes (Amaducci et al., 2012). The vegetative growth period is characterized by biomass production, after which this behavior shifts toward fiber maturation (secondary fiber formation and lignification) and plant reproduction during flowering development (Van Der Werf and Turunen, 2008; Liu et al., 2015). At northern latitudes, the shift from long-day toward short-day photoperiod regimes occurs later and therefore the critical photoperiod for flowering (~14–16 h) is reached later in the growing season of hemp (Struik et al., 2000; Hall et al., 2012). As a result, the cultivation of hemp focused on the production of fibers may be better in northern latitudes, while the cultivation of hemp for seeds or dual purpose seed/fiber in southern latitudes may be more adapted to the environment (Amaducci et al., 2015). The selection of the cultivation purposes based on the environment might increase the profitability of hemp cultivation, complementing the high fiber quality achieved by the breeding programs using the heritable phenotypic variation.

Another example of the adaptive behavior of hemp's biomass is the difference in production of monosaccharides composing pectin from plants across environments. Plants grown in France showed the largest content of monosaccharides composing pectin while plants of the same accessions in the Netherlands showed the lowest contents. It has been previously reported that pectin plays a role in modulating cell wall architecture in response to low availability of water [reviewed in Le Gall et al. (2015)], owing to its water holding capacity function (Voragen et al., 2009). As shown in **Table 2**, France had lower rainfall and larger days without rain than Netherlands. Based on the environmental differences between the locations and the functions of pectin, it seems plausible to hypothesize that hemp plants may increase the content of pectin in the stem partially as a response to the changes in water availability. This relationship may have important implications in the improvement of the fiber quality of hemp, as the contents of monosaccharides composing pectin are poorly heritable traits and pectin plays a key role in the fiber quality. Pectin has been associated to difficulties in decortication which results in increased fiber damage (Ranalli, 2004; Müssig et al., 2008; Salentijn et al., 2015) after fiber decortication. Furthermore, plants grown in Netherlands showed larger decortication efficiencies than in the other two locations, and the content of galacturonic acid, the main component of pectin (Willats et al., 2001), was positively correlated to the shive content after decortication (χ). These results indicate that lower contents of galacturonic acid in the stems results in easier decortication. In addition, it suggests that water availability may play a role in the pectin content, and if that is the case, it could be used as a tool to improve fiber quality associated to poor heritable traits. Crop management, such as irrigation, could contribute to the decrease in the pectin content and thus improve the quality. The present study provides interesting results to further investigate the influence of water availability on pectin content of hemp. The use of a wider range of locations with contrasting and more detailed environmental conditions and the use of controlled experiments may be useful to get insights into the role of specific environmental factors in hemp fiber quality.

Finally, the correlation analysis revealed that monoecious and dioecious plants have a different relationship with fiber quality. The results of the hemp panel analysis confirm that monoecious accessions have larger fiber qualities than dioecious. These differences may be explained by the larger uniformity in fiber production common in monoecious accessions compared to dioecious accessions (Mandolino and Carboni, 2004; Amaducci and Gusovius, 2010; Faux et al., 2013; Faux et al., 2014; Salentijn et al., 2015; Faux et al., 2016). Finally, the sex determination of hemp is another key element that should be taken into account when selecting the germplasm for breeding programs as it has important implications in fiber quality (Amaducci and Gusovius, 2010; Amaducci et al., 2015).

### Implications of the Fiber Quality Variability in the Development of New Hemp Cultivars With Improved Fiber Properties

In the present study, the extent of fiber quality variation among accessions reveals a good hemp panel to further study the genetic architecture of fiber quality, flowering, and sex characteristics of hemp. As Davis (2008) described in a previous study, in order to genetically improve some traits, they must be heritable. The contents of most cell wall components [glucose, mannose, xylose, glucuronic acid, and lignin (ADL and KL)], content of bast fiber, flowering time measurements, and sex determination of hemp have high heritability values, meaning that they are good candidates for genetic association studies. The selection of plants harboring favorable alleles for these traits would maximize the genetic gains expected from the breeding programs aiming to increase the quality of the bast fiber. However, traits with low genetic components and relatively large *G*×*E* interactions, such as monosaccharides composing pectin, are not appropriate candidates for mapping studies, as the statistical tools currently available have low power to discriminate between phenotypic variations owing to genetic or environmental effects, when the genetic components are small. As a result, the statistical power of the association for these traits would drop, leading to high false-positives and/or false-negative rates (Huang and Han, 2014; Bernardo, 2016). Yet, the large adaptive behavior of these traits suggests that crop management practices may be a good alternative to breeding for traits poorly heritable. Therefore, the combination of breeding programs to target traits with large genetic components and crop management for traits with small genetic component may be a good strategy to improve the potential of hemp as a high-yielding, sustainable crop of excellent fibers.

## Conclusions

The results of this study prescribe positive prospects for the development of new hemp cultivars with improved fiber quality properties. In particular, the hemp accession panel reveals to be a good dataset for mapping studies owing to the extensive phenotypic variability of 28 fiber quality-related traits. The content of most cell wall components (cellulose, hemicellulose, and lignin), bast fiber content, flowering time traits, and sex determination showed large heritable variation, controlled by robust genetic mechanisms that can be used in breeding programs. In addition, all traits showed statistically significant *G*×*E* interaction components in different percentages depending on the traits. These results suggest that the phenotypic variation in fiber quality of hemp has a fraction of heritable variation sensitive to the environment, controlled by plastic genetic mechanisms.

In addition, fiber quality traits were strongly affected by the environment, such as photoperiod and temperature regimes and probably water availability. These sensitivities can be understood as adaptations to the environment, independently of the heritable genetic variation. The adaptive behavior of poorly heritable traits, such as pectin, might be used to develop strategies, such as crop management practices, to increase fiber quality alternatively to breeding programs. Finally, the correlation analysis revealed that monoecious plants have larger fiber quality than dioecious hemp owing to probably uniformity in fiber production common in monoecious accessions. Altogether, we advocate for novel hemp breeding programs that breed for highly heritable traits, taking into account the sex determination of the germplasm in the breeding schemes and considering the environmental sensitivity of fiber quality.

## Data Availability Statement

All datasets generated for this study are included in the article/**Supplementary Material**.

## Author Contributions

JP designed and performed the experiments, analyzed the data, and wrote the manuscript. ES helped designing and performing the experiments, helped analyzing the data, and revised the manuscript. M-JP helped analyzing the data and revised the manuscript. CT, BD, and GM performed field trials and revised the manuscript. H-JG, KT, SA, SW, BU, and JM performed experiments and revised the manuscript. LT coordinated and supervised this study, defined the experimental strategy, discussed the outcomes, and revised the manuscript.

## Funding

This work was conducted as part of the MultiHemp project (Multipurpose hemp for industrial bioproducts and biomass) funded by the European Union's Seventh Framework Programme for research, technological developments, and demonstration under grant agreement number 311849.

## Conflict of Interest

The authors declare that the research was conducted in the absence of any commercial or financial relationships that could be construed as a potential conflict of interest.
