# Supplementary material for: Genetic Variability of Morphological, Flowering, and Biomass Quality Traits in Hemp (Cannabis sativa L.)
Source: Front Plant Sci. 2020 Feb 20;11:102. doi: 10.3389/fpls.2020.00102 (PMC7044243; doi:10.3389/fpls.2020.00102)
Supplement: Supplementary file 1 [file DataSheet_1.docx]

**Supplementary Material:**

**Materials and Methods - Biochemical analysis of hemp cell walls: NIRS model development**

The models were externally validated with 24 randomly selected samples. Biochemical analysis of monosaccharide composition and content of Klason lignin were performed according to (Petit et al., 2019) and acid detergent lignin was analysed according to the protocol developed by Ankom Technology (ANKOM Technology Corporation, Fairpoint, NY) based on the previous established protocols (Goering and van Soest, 1970). Biochemical and NIRS data from model and validation samples were used to develop the prediction models and to validate them, using WinISI version 4.9 statistical software (Foss, Hillerød, Denmark). The prediction equations were generated using modified partial least square regression analysis (Shenk and Westerhaus, 1991) with 1:4:4:1 as parameters for derivation and smoothing of NIRS. The first 7 principal components were manually selected for modelling the equations which included 98.76% of the NIRS variation. The quality of the prediction models was evaluated with an internal cross-validation and with an external validation. The cross-validation of the models was evaluated by calculating the squared Pearson coefficient of correlation (r^2^) and the standard error of the cross-validation (SECV) between the biochemical and the predicted data of the model samples. The external validation was evaluated by calculating the r^2^ between the biochemical and the predicted data of the external validation samples and by comparing for these samples the standard error of the prediction (SEP) and the standard error of laboratory (SEL) for each trait. **Tables 1** and **2** detail internal cross-validation and external validation statistics of the prediction models. The models were used to predict the cell wall composition of 1034 stem samples.

**Supplementary Table 1.** Summary of internal cross-validation statistics of mPLS models used for the prediction of cell wall composition. See **Table 3** of the paper for abbreviations.

| Trait | Number of samples | Biochemical data | | | NIRS predicted data | | | *r^2^* | SEC | SECV |
| --- | --- | --- | --- | --- | --- | --- | --- | --- | --- | --- |
|  |  | Mean | Min | Max | Mean | Min | Max |  |  |  |
| ADL%dm | 112 | 9.23 | 6.55 | 14.13 | 9.19 | 5.07 | 13.30 | 0.90 | 0.43 | 0.53 |
| Ara%dm | 110 | 0.74 | 0.27 | 1.41 | 0.72 | 0.00 | 1.45 | 0.94 | 0.06 | 0.08 |
| Gal%dm | 112 | 1.45 | 0.74 | 2.55 | 1.45 | 0.33 | 2.57 | 0.89 | 0.12 | 0.13 |
| GalA%dm | 112 | 5.15 | 3.89 | 8.61 | 5.07 | 2.26 | 7.88 | 0.91 | 0.27 | 0.38 |
| GlcA%dm | 115 | 0.39 | 0.17 | 0.75 | 0.39 | 0.08 | 0.70 | 0.71 | 0.06 | 0.07 |
| Glc%dm | 114 | 49.34 | 42.38 | 58.56 | 49.22 | 37.83 | 60.61 | 0.75 | 1.89 | 2.06 |
| KL%dm | 114 | 14.97 | 9.812 | 20.53 | 14.96 | 8.77 | 21.15 | 0.87 | 0.79 | 0.87 |
| Man%dm | 116 | 2.63 | 1.69 | 4.09 | 2.63 | 1.16 | 4.10 | 0.80 | 0.22 | 0.28 |
| Rha%dm | 112 | 0.82 | 0.62 | 1.13 | 0.82 | 0.56 | 1.08 | 0.76 | 0.04 | 0.05 |
| Xyl%dm | 112 | 14.10 | 9.53 | 18.70 | 14.10 | 6.99 | 19.70 | 0.91 | 0.55 | 0.69 |

r^2^ = squared Pearson coefficient of correlation. SEC = standard error of the calibration. SECV = standard error of the cross-validation

**Supplementary Table 2.** Summary of external validation statistics of mPLS models used for the prediction of cell wall composition. See **Table 3** for abbreviations.

| Trait | Number of samples | Biochemical data | | | NIRS predicted data | | | Slope | Intercept | *r^2^* | SEP | SEL |
| --- | --- | --- | --- | --- | --- | --- | --- | --- | --- | --- | --- | --- |
|  |  | Mean | Min | Max | Mean | Min | Max |  |  |  |  |  |
| ADL%dm | 24 | 8.79 | 6.84 | 12.64 | 9.06 | 7.74 | 12.35 | 1.14 | -0.26 | 0.91 | 0.47 | 0.25 |
| Ara%dm | 24 | 0.83 | 0.42 | 1.18 | 0.80 | 0.45 | 0.93 | 1.05 | 0.02 | 0.89 | 0.07 | 0.04 |
| Gal%dm | 24 | 1.66 | 0.91 | 2.31 | 1.53 | 0.98 | 1.67 | 1.45 | 0.12 | 0.81 | 0.23 | 0.08 |
| GalA%dm | 24 | 5.23 | 3.99 | 6.08 | 5.17 | 4.06 | 5.60 | 0.96 | 0.06 | 0.81 | 0.35 | 0.16 |
| GlcA%dm | 24 | 0.33 | 0.52 | 0.35 | 0.35 | 0.50 | 0.32 | 0.94 | -0.02 | 0.75 | 0.05 | 0.02 |
| Glc%dm | 24 | 50.91 | 47.96 | 50.4 | 49.30 | 46.80 | 48.71 | 0.62 | 1.64 | 0.22 | 3.54 | 0.70 |
| KL%dm | 24 | 14.51 | 11.68 | 17.44 | 14.50 | 14.90 | 17.33 | 0.86 | 0.05 | 0.60 | 0.91 | 0.29 |
| Man%dm | 24 | 2.90 | 2.15 | 2.52 | 2.86 | 2.30 | 2.68 | 1.14 | 0.05 | 0.74 | 0.25 | 0.10 |
| Rha%dm | 24 | 0.87 | 0.71 | 1.03 | 0.84 | 0.72 | 0.90 | 1.38 | 0.02 | 0.82 | 0.05 | 0.02 |
| Xyl%dm | 24 | 13.93 | 17.07 | 14.17 | 13.90 | 16.68 | 13.91 | 0.94 | 0.03 | 0.71 | 0.76 | 0.30 |

r^2^ = squared Pearson coefficient of correlation. SEP = standard error of the prediction. SEL = Standard error laboratory

**Supplementary Table 3.** Ranges of statistical differences between the three locations for 28 fibre quality traits. This information corresponds to the data presented in **Figure 1.** See **Table 3** for abbreviations.

| Trait | Location | | |
| --- | --- | --- | --- |
|  | CRA | FNPC | VDS |
| ADL (%) | a | a | b |
| Ara (%) | b | c | a |
| Gal (%) | b | c | a |
| GalA (%) | b | c | a |
| Glc (%) | b | a | b |
| GlcA (%) | a | b | c |
| KL (%) | a | a | b |
| Man (%) | c | b | a |
| Rha (%) | b | c | a |
| Xyl (%) | b | a | c |
| D (mm) | b | a | c |
| H (cm) | b | a | c |
| DW (%) | a | a | b |
| DW_S (%) | c | b | a |
| DW_L5 (g) | b | a | c |
| M0 (%) | b | a | c |
| MF0 (g) | b | a | b |
| MF1 (g) | b | a | b |
| BCD (%) | b | b | a |
| nDec_1 (%) | a | b | c |
| nDec_2 (%) | a | a | b |
| X (%) | b | b | a |
| PL (mm water) | a | c | b |
| PH (mm water) | a | c | b |
| FL_Begin (∑Cd) | a | b | b |
| FL_Full (∑Cd) | b | ab | a |
| VEG (days) | a | b | c |
| Sex_det | a | a | a |


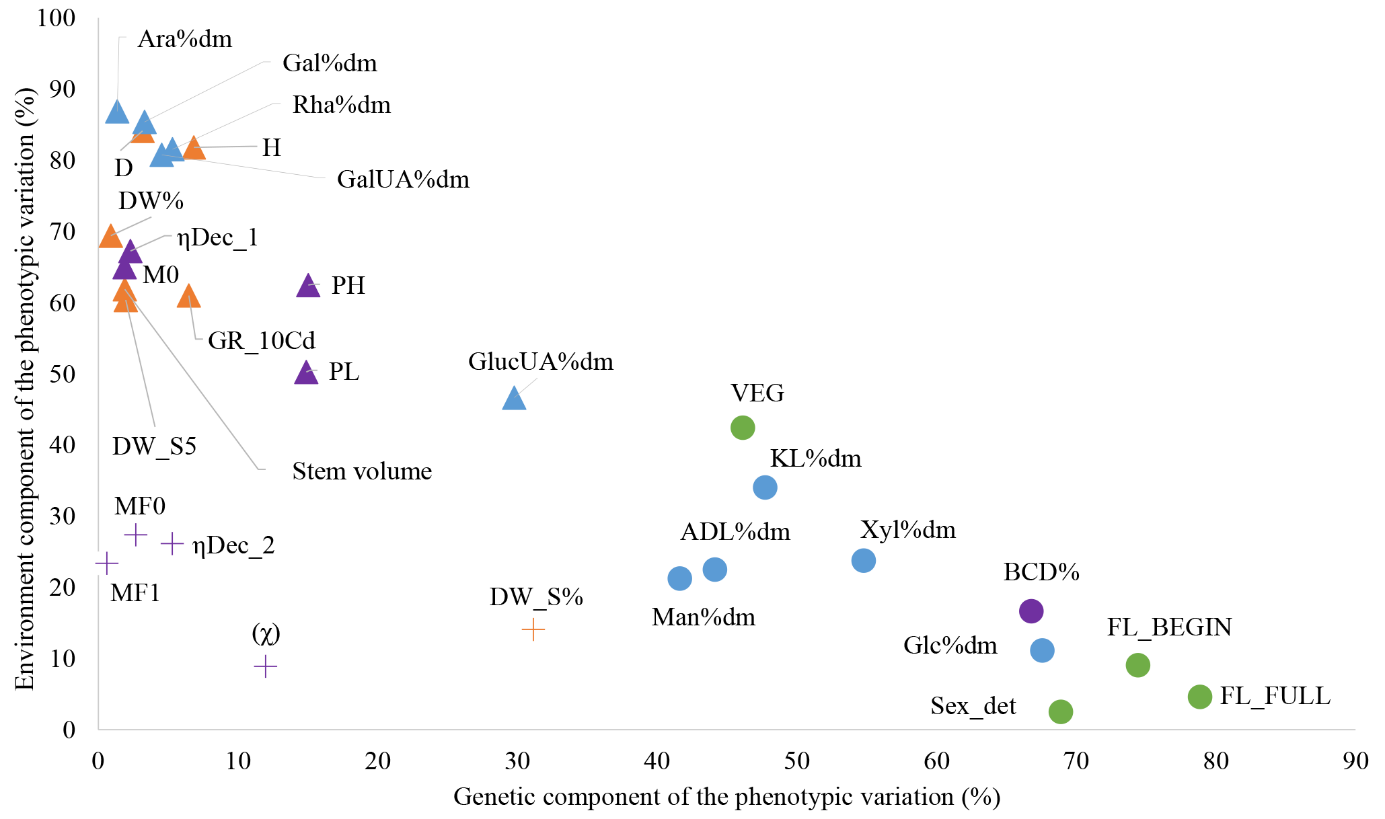


**Supplementary Figure 1.** Scatterplot of the phenotypic variation of 30 traits explained by the percentage of genetic (x-axis) and environment (y-axis) components. The colour of the markers indicate the type of trait: orange, green, purple and blue represent agronomic parameters, flowering/sex stages, fibre measurements and cell wall traits respectively. The shape of the markers indicate the pattern of the phenotypic variation predominant in each trait: circle, triangle and cross represent genetic, environment and residual main component respectively. See **Table 3** for abbreviations.

**Materials and Methods - Network analysis:**

The most important correlations were represented with a network analysis based on graphical lasso performed in R, using the High-Dimensional Undirected Graph Estimation (HUGE) package version 1.2.7 (Zhao et al., 2012). The best solution, using the StARS criterion at α = 1 (Liu et al., 2010), was non-informative. Several lambdas were used and an intermediate solution was found at λ = 0.51. Boxplots were also graphed in R using ggplot2 package.


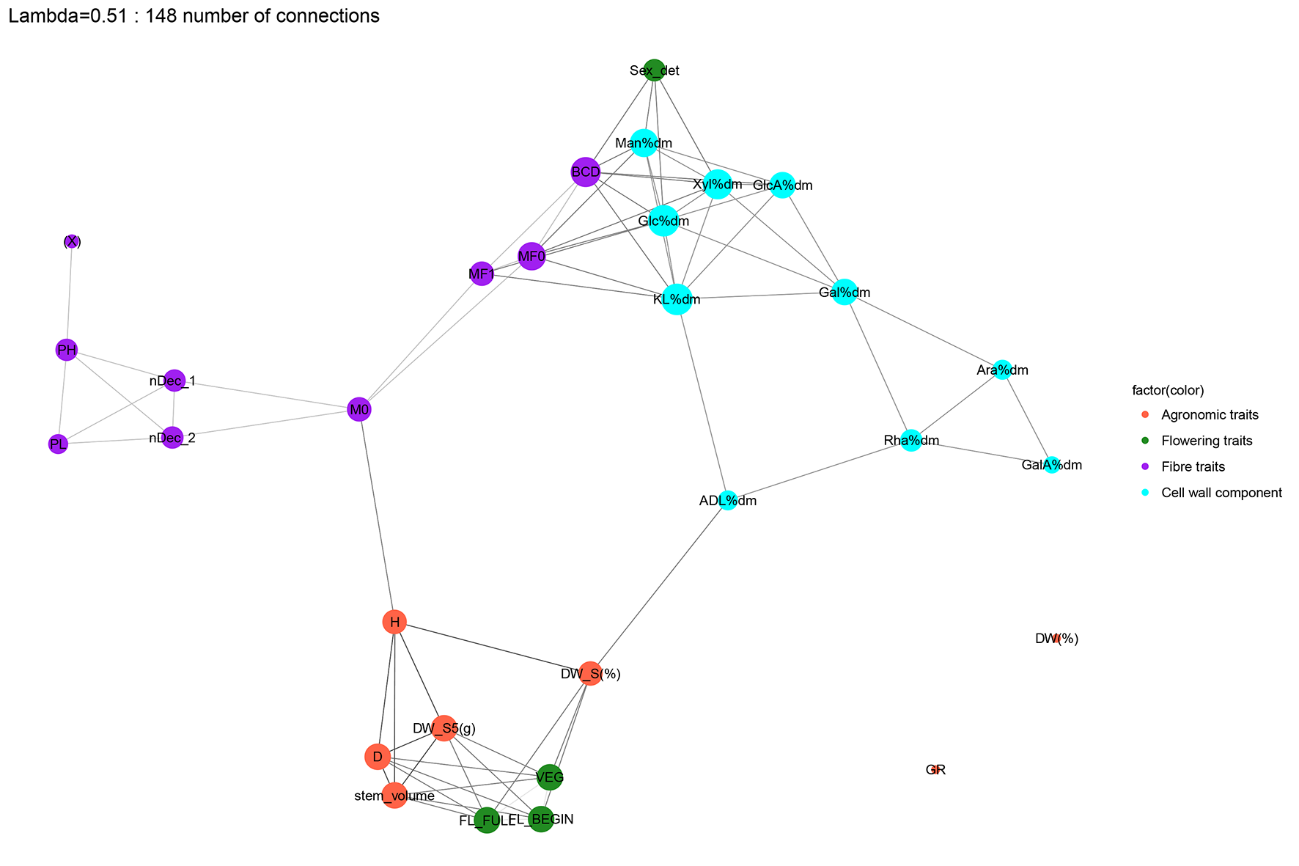


**Supplementary Figure 2.** Network analysis based on graphical lasso of 30 hemp traits including agronomic parameters, flowering stages, fibre traits and cell wall components at λ = 0.51. See **Table 3** for abbreviations.

**References Supplementary Material:**

Goering, H.K., and Van Soest, P.J. (1970). "Forage fiber analyses (apparatus, reagents, procedures, and some applications)". (Washington, D.C.: Agricultural Research Service, U.S. Dept. of Agriculture).

Liu, H., Roeder, K., and Wasserman, L. 2010. Stability Approach to Regularization Selection (StARS) for High Dimensional Graphical Models. *arXiv e-prints* [Online]. [Accessed June 01, 2010].

Petit, J., Gulisano, A., Dechesne, A., and Trindade, L.M. (2019). Phenotypic Variation of Cell Wall Composition and Stem Morphology in Hemp (*Cannabis sativa* L.): Optimization of Methods. *Front. Plant Sci.* 10. doi: 10.3389/fpls.2019.00959.

Shenk, J.S., and Westerhaus, M.O. (1991). Populations Structuring of Near Infrared Spectra and Modified Partial Least Squares Regression. *Crop Sci.* 31**,** 1548-1555. doi: 10.2135/cropsci1991.0011183X003100060034x.

Zhao, T., Liu, H., Roeder, K., Lafferty, J., and Wasserman, L. (2012). The huge Package for High-dimensional Undirected Graph Estimation in R. *J. Mach. Learn. Res.* 13**,** 1059-1062.
